# Characteristics of Human Turbinate-Derived Mesenchymal Stem Cells Are Not Affected by Allergic Condition of Donor

**DOI:** 10.1371/journal.pone.0138041

**Published:** 2015-09-16

**Authors:** Se Hwan Hwang, Hye Kyung Cho, Sang Hi Park, WeonSun Lee, Hee Jin Lee, Dong Chang Lee, Sun Hwa Park, Mi Hyun Lim, Sang A Back, Byeong Gon Yun, Dong Il Sun, Jun Myung Kang, Sung Won Kim

**Affiliations:** 1 Department of Otolaryngology-Head and Neck Surgery, The Catholic University of Korea, College of Medicine, Seoul, Korea; 2 Department of Pediatrics, Graduate School of Medicine, Gachon University, Incheon, Korea; 3 Institute of Clinical Medicine Research, College of Medicine, Catholic University of Korea, Seoul, Korea; 4 Department of biomedical science, The Catholic University of Korea, College of Medicine, Seoul, Korea; Faculty of Animal Sciences and Food Engineering, University of São Paulo, BRAZIL

## Abstract

The characteristics of mesenchymal stem cells (MSCs) derived from human turbinates (hTMSCs) have not been investigated in allergic rhinitis. We evaluated the influence of allergic state of the donor on the characteristics, proliferation, and differentiation potential of hTMSCs, compared with hTMSCs derived from non-allergic patients. hTMSCs were isolated from five non-allergic and five allergic patients. The expression of toll-like receptors (TLRs) in hTMSCs was measured by FACS, and cell proliferation was measured using a cell counting kit. Cytokine secretion was analyzed using multiplex immunoassays. The osteogenic, chondrogenic, and adipogenic differentiation potentials of hTMSCs were evaluated by histology and gene expression analysis. In allergic patients, FACS analysis showed that TLR3 and TLR4 were more highly expressed on the surface of hTMSCs than TLR2 and TLR5. The proliferation of hTMSCs was not influenced by the presence of TLR priming. The expression of IL-6, IL-8, IL-12, IP-10, and RANTES was upregulated after the TLR4 priming. The differentiation potential of hTMSCs was not influenced by TLR priming. These characteristics of hTMSCs were similar to those of hTMSCs from non-allergic patients. We conclude that the allergic condition of the donor does not influence TLR expression, proliferation, or immunomodulatory potential of hTMSCs.

## Introduction

Rhinitis is a heterogeneous disease featured by one or more of the following nasal symptoms: sneezing, rhinorrhea, and nasal obstruction. Approximately 50% of rhinitis cases are caused by allergy (allergic rhinitis) [1]. Allergic rhinitis (AR) is induced by an immunoglobulin E (IgE)-mediated immune response to certain allergens in nasal mucosa [2] that involves the release of inflammatory mediators and the activation and recruitment of cells to the nasal mucosa [1]. Nasal obstruction in rhinitis is usually related to hypertrophy of the inferior turbinates. In these cases, surgical reduction of inferior turbinates, such as partial turbinectomy, can be offered [3]; surgery of the turbinates is, in fact, very common and represents the eighth most common procedure performed in otorhinolaryngologic surgery in order to increase the nasal airflow [4].

Mesenchymal stem cells (MSCs) have the potential to differentiate into chondrogenic, osteogenic, adipogenic, and neurogenic cells, as well as possessing immunomodulatory properties. Studies have shown that MSCs exist in diverse tissues and organs, including nervous tissue, skin, muscles, and adipose tissue. Human MSCs show differences that influence their functional attributes depending on the tissue from which they are derived [5]. Previously, we have isolated human turbinate-derived mesenchymal stem cells (hTMSCs) from human inferior turbinate discarded during partial turbinectomy, and demonstrated that their properties relating to proliferation, differentiation, immunomodulation, and the effects of cell passage and donor age, differ from those of bone marrow-derived mesenchymal stem cells (BM-MSCs) and adipose-derived mesenchymal stem cells (AD-MSCs) [6–10]. However, we did not address whether the characteristics of hTMSCs were affected by the allergic condition of the donor, despite the high proportion of AR in rhinitis. The mucosal surfaces of nasal cavity persistently contact large amounts of allergen, and the activation of an immune response against an allergen may alter the characteristics of MSCs derived from respiratory mucosa in allergic rhinitis. Therefore, it is important to understand the characteristics of hTMSCs originating from allergic patients. In this study, we aimed to determine whether hTMSC proliferation, differentiation, and immunomodulatory function were influenced by allergic state.

## Materials and Methods

All studies utilizing hTMSCs were conducted after written approval (HC13TISI0038) from the Institutional Review Board of the Catholic Medical Center Clinical Research Coordinating Center and after obtaining written informed consent from the donors themselves. Investigations were conducted in accord with the principles expressed in the Declaration of Helsinki. Inferior turbinate tissue was obtained from 10 patients over the age of 20 undergoing partial turbinectomy (five patients with allergic rhinitis and five patients with non-allergic rhinitis). The presence or absence of allergic rhinitis was diagnosed based on clinical symptoms and the detection of serum specific IgE (multiple allergen simultaneous test). Patients with sinusits, nasal polyposis, or immunologic problems were excluded.

### Cell isolation and TLR priming protocol

For each patient, the equal amount (0.0366 g) of turbinate tissue was gained from tissue removed during partial turbinectomy. hTMSCs were isolated as previously described [6] and examined, after four passages, for toll-like receptor (TLR) agonist activation-related changes in immunophenotype, proliferation, and multipotent differentiation.

In the TLR-priming protocol [11, 12], hTMSCs grew to 60–70% confluent in culture medium (DMEM containing 10% FBS) before the beginning of each experiment. LPS (10 ng/ml, Sigma-Aldrich, St. Louis, MO) and poly(I:C) (1 mg/ml, Sigma-Aldrich) were added to fresh growth medium as the hTMSCs agonists for TLR4 or TLR3, individually, and incubated with hTMSCs for 1 hr. The cells were washed twice in growth medium without TLR agonists and measured as described for each experiment.

### Characterization of immunophenotype on hTMSCs

For the measurements of cell surface markers via flow cytometry, the hTMSCs were plated at a density of 1 × 10^5^ cells/ml into a test tube (BD, Franklin Lakes, NJ) and washed three times with wash buffer (PBS with 3% FBS) as previously described [12]. The antibodies against CD14 (all anti-human CD from BD Biosciences, San Jose, CA), CD19, CD34, CD73, CD90, CD105, HLA-DR, TLR 2 (ab9100) (all anti-human TLR from Abcam, Cambridge), TLR 3 (ab12085), TLR 4 (ab30667), and TLR 5 (ab13875) were added to the incubation of the hTMSCs as the primary antibody. Cell fluorescence was evaluated by flow cytometry using a FACS-Calibur instrument (BD).

### Cytokine assays

hTMSCs were plated at a density of 1 × 10^5^ in 24-well plates, allowed to adhere overnight, and pre-treated with TLR3 or TLR4 agonists for 1 hr as previously described [12]. IL (interleukin)-1α, IL-1β, IL-4, IL-6, IL-8, IL-10, IL-12, IP-10 (CXCL10), RANTES (CCL5), TNF-α, GM-CSF, and IFN-γ were analyzed with the Milliplex Map human cytokine/chemokine multiplex immunoassay (Millipore, Billerica, MA). These experiments were conducted at least three times on each hTMSCs pools.

### Proliferation assay

hTMSCs were pretreated with TLR3 or TLR4 agonists for 1 hr and plated concurrently into 96-well tissue culture plates at a density of 1500 cells per well as previously described [12]. Cell proliferation was measured for seven days using a cell counting kit (Cell Counting Kit-8; CCK-8) (Dojindo Laboratories, Kumamoto, Japan) and determined at least in triplicate.

### Multilineage differentiation potential of hTMSCs

Trilineage-differentiation (osteogenesis, chondrogenesis, and adipogenesis) were induced as described previously [12]. For the evaluation of the osteogenesis, hTMSCs were assessed histologically with alkaline phosphatase (ALP) staining and by RT-PCR second weeks after the induction cultures. For the evaluation of the chondrogenesis, hTMSCs were assessed for histologically with toluidine blue staining and RT-PCR second weeks after induction culture. For the evaluation of the adipogenesis, hTMSCs were assessed histologically with Oil Red O staining and by RT-PCR second weeks after the induction cultures.

### RNA extraction and RT-PCR of hTMSCs

RNA was isolated and cDNA synthesized as described previously [12]. Quantitative expression was determined by using Real-time PCR using TaqMan gene expression assays (Applied Biosystems, Foster City, CA) for Runt-related transcription factor 2 (RUNX2), type I collagen, type II collagen, AcylCoA synthetase (ACS), and peroxisome proliferator-activated receptor γ (PPARγ) (Table 1). Glyceraldehyde 3-phosphate dehydrogenase served as an endogenous control.

**Table 1:** Gene expression assays used for real-time polymerase chain reaction for Multilineage differentiation.

Gene	Abbreviation	Reference Sequence	Assay number
type I collagen	COL1	NM_000088	Hs00164004_m1
runt-related transcription factor 2	Runx2	NM_001015051	Hs00231692_m1
type II collagen	COL2	NM_001844	Hs00264051_m1
peroxisome proliferator-activated	PPARγ	NM_138712	Hs01115513_m1
receptor gamma			
acyl-CoA synthetase short-chain family	ACS	NM_001076552	Hs00218766_m1
member 2			
Glyceraldehyde 3-phosphate	GAPDH	NM_002046	Hs99999905_m1

### Statistical analysis

T-test and one-way analysis of variance (ANOVA) were used to test for statistical significance of the differences between groups and a *p* value<0.05 was considered statistical significant.

## Results

### Characterization of TLR expression pattern in hTMSCs derived from allergic and non-allergic patients by flow cytometry

**Fig 1:**
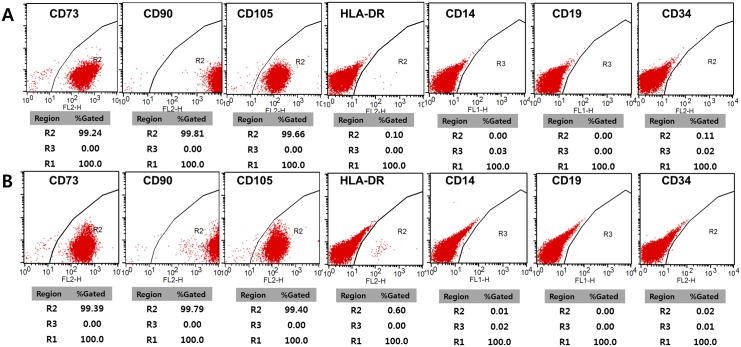
Fluorescence-activated cell sorting (FACS) analysis of human turbinate-derived mesenchymal stem cells (hTMSCs) from nonallergic patients (A) and allergic patients (B). FACS after three passages was used to identify hTMSCs from both groups as positive for CD73, CD90, and CD105, and negative for CD14, CD19, CD34, and HLA-DR.

Cell surface markers of in vitro-cultured hTMSCs were examined by flow cytometric analysis to measure the expression of several mesenchymal stem cell markers, HLA antigens, and hematopoietic stem cell markers. hTMSCs from allergic patients were negative for hematopoietic markers (CD14, CD19, CD34, and HLA-DR) and positive for MSC markers (CD73, CD90, and CD105) (Fig 1).

**Fig 2:**
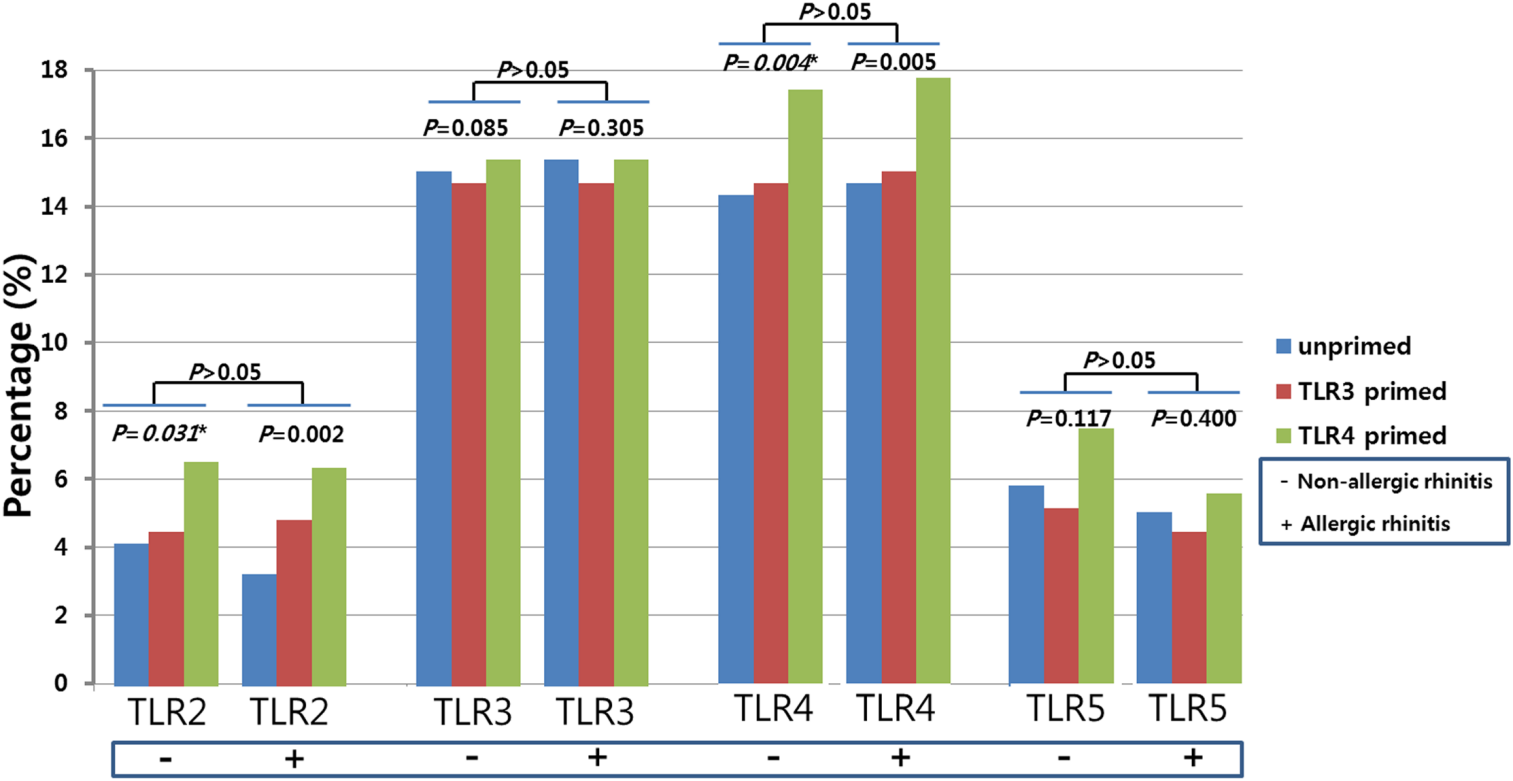
Basal toll-like receptor (TLR) expression and response to TLR agonists in human turbinate-derived mesenchymal stem cells (hTMSCs) from allergic and non-allergic patients. Flow cytometry analysis demonstrated that expression of TLR3 and TLR4 in hTMSCs was higher than that of TLR2 and TLR5. Cells were unprimed or primed with poly(I:C) (TLR3 agonist), or LPS (TLR4 agonist). LPS significantly affected the expression of TLR2 and TLR4. These results were similar to those of hTMSCs from non-allergic patients.

Measurement of TLR protein expression such as TLR2, TLR3, TLR4, and TLR5 in hTMSCs from allergic patients was achieved by flow cytometry. Flow cytometry analyses indicated that TLR3 and TLR4 were more highly expressed on the surface of hTMSCs than TLR2 and TLR5 (Fig 2). Stimulation of the hTMSCs from allergic patients with TLR4 or TLR3 resulted in different levels of TLR expression in flow cytometry. Consistent with our published results [1], TLR Priming (TLR4 or TLR3) did not significantly influence the expression of TLR3 or TLR5, but TLR4 priming significantly upregulated the expression of TLR2 and TLR4. Overall, the basal expression levels of TLRs, and response to TLR priming, were similar to those of hTMSCs derived from non-allergic patients (Fig 2).

### Cytokine assay according to priming of TLR in hTMSCs derived from allergic and non-allergic patients

**Fig 3:**
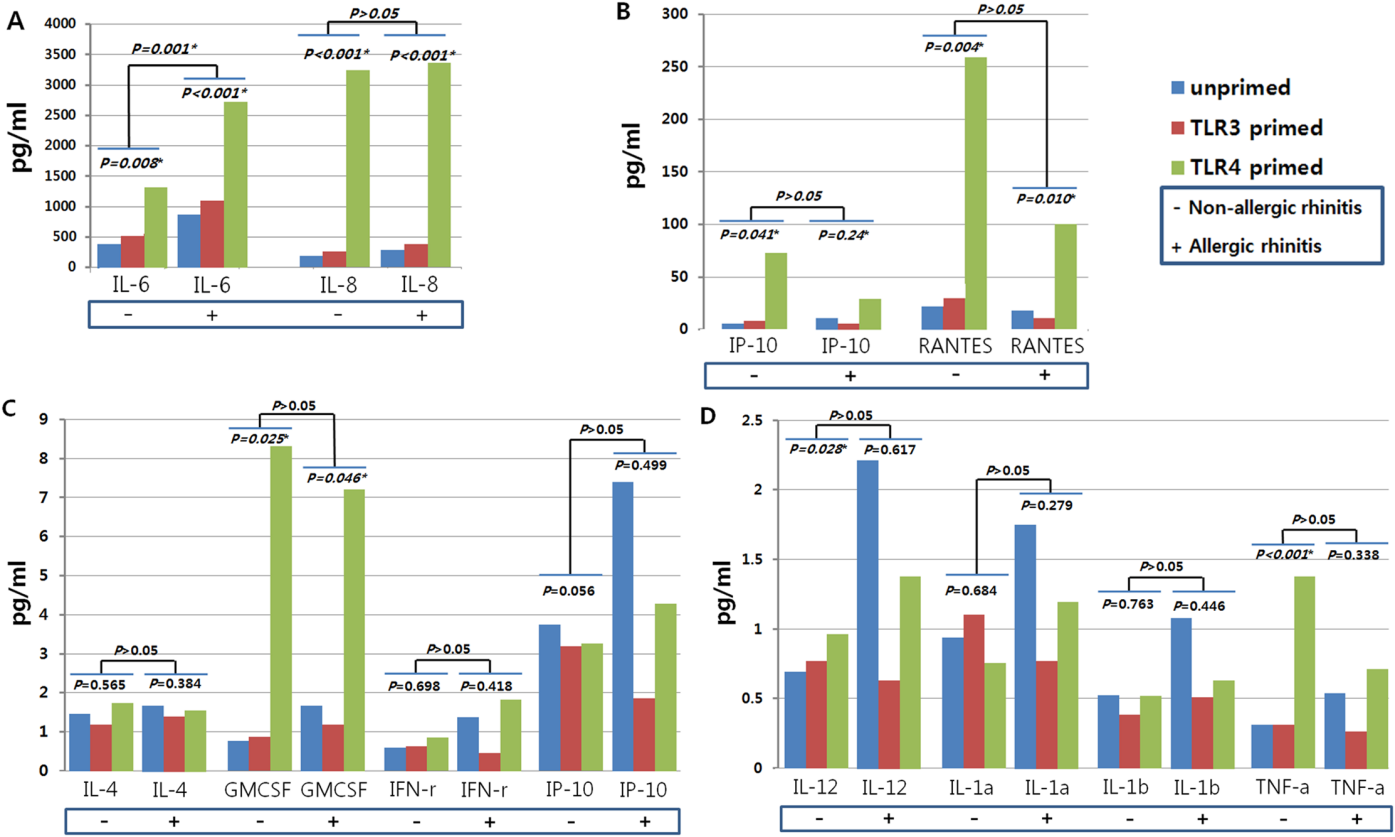
Effects of toll-like receptor 3 (TLR3) and TLR4 agonists on cytokine secretion by human turbinate-derived mesenchymal stem cells (hTMSCs) from allergic and non-allergic patients. Secretion of the cytokines and chemokines IL-1α, IL-1β, IL-4, IL-6, IL-8, IL-10, IL-12, IP-10 (CXCL10), RANTES (CCL5), TNF-α, GM-CSF, and IFN-γ by hTMSCs after exposure to TLR3 and TLR4 agonists was assessed by multiplex immunoassay. In the hTMSCs of allergic patients, LPS strongly induced expression of IL-6, IL-8, IP-10 (CXCL10), RANTES (CCL5), and GM-CSF, while poly(I:C) did not influence the secretion of cytokines. These secretion patterns of cytokines were similar to those of hTMSCs from non-allergic patients.

We intended to extend our previous results of the effect that TLR priming has on the immune modulating property of hTMSCs, as well as to provide an explanation for the effect of allergic statue of donor on this property. IL-1α, IL-1β, IL-4, IL-6, IL-8, IL-10, IL-12, IP-10 (CXCL10), and RANTES (CCL5) were detected with mean values of higher than 1 pg/ml in supernatants of hTMSCs derived from allergic patients under unprimed condition. These cytokines and chemokines was not significantly different from those in supernatants of hTMSCs derived from non-allergic patients, although levels of all analytes, except RANTES (CCL5), tended to be higher in allergic patients than in non-allergic patients (Fig 3).

To investigate the responsiveness of hTMSCs derived from allergic patients to the exposure of TLR agonists, we exposed the cells to LPS or poly(I:C). The TLR4 priming highly increased expression of IL-6, IL-8, GM-CSF, IP-10 (CXCL10), and RANTES (CCL5), whereas the TLR3 priming had no effect on the expression of these cytokines. An approximate 3–10-fold increase in IL-6, IL-8, GM-CSF, IP-10 (CXCL10), and RANTES (CCL5) was detected following the TLR 4 priming (Fig 3). Overall, basal cytokine and chemokine levels, and response to TLR priming, in hTMSCs derived from allergic patients, were similar to those of hTMSCs derived from non-allergic patients with the exception of increased expression of IL-12 and TNF-α following TLR4 priming in hTMSCs from the non-allergic group.

### Proliferation assay of hTMSCs derived from allergic and non-allergic patients

**Fig 4:**
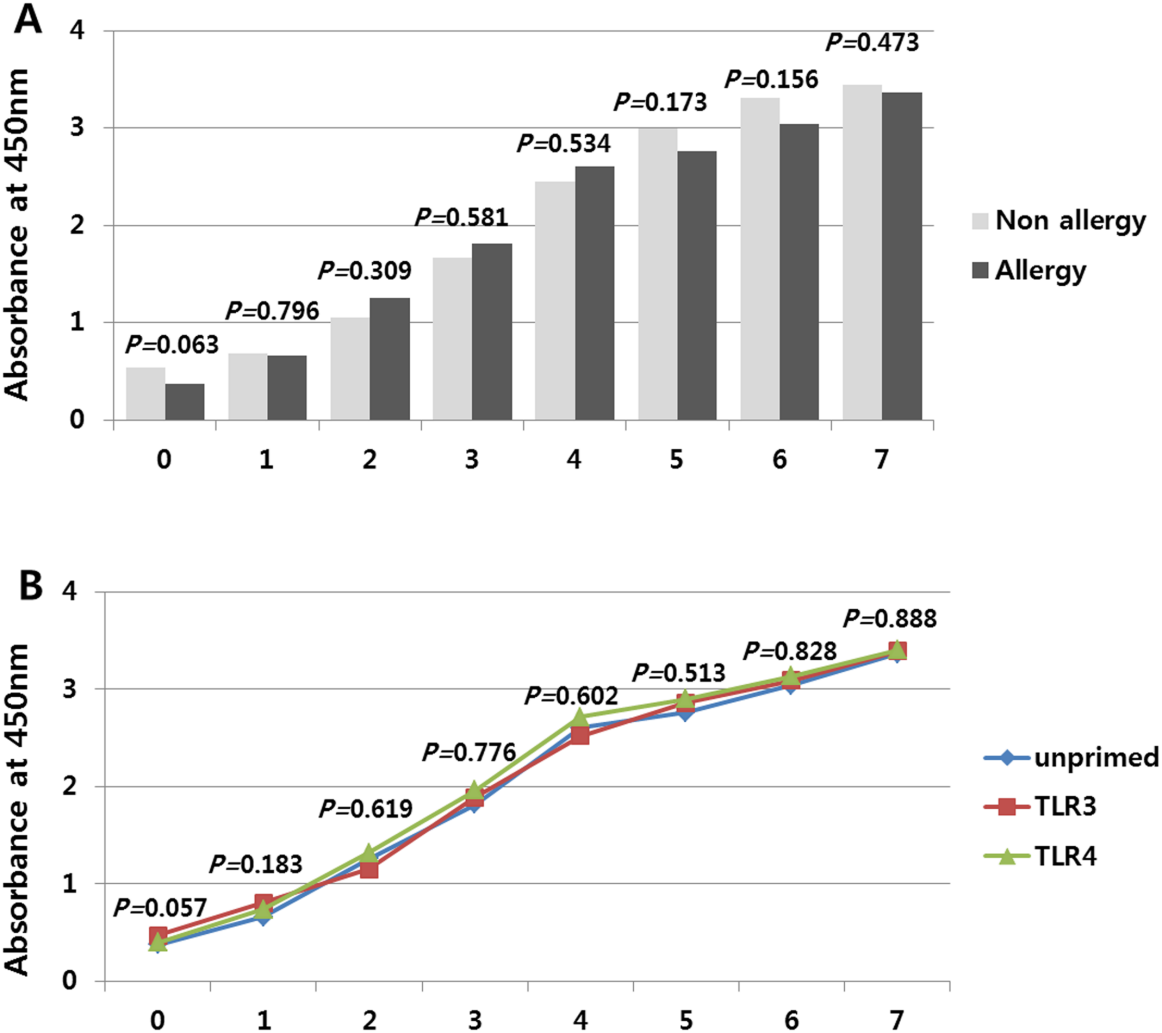
Cell proliferation with and without toll-like receptor (TLR) agonist treatment in human turbinate-derived mesenchymal stem cells (hTMSCs) from allergic and non-allergic patients. Cell proliferation was monitored for a period of seven days. hTMSCs from allergic patients exhibited rapid proliferation from days two to four. The proliferation patterns were similar to those of MSCs derived from non-allergic patients. Exposure to TLR agonists had no effect on the proliferation of hTMSCs from allergic patients.

To examine whether the allergic condition of donor and TLR activation influences the proliferation of hTMSCs, the pattern of cell proliferation was monitored during seven days. hTMSCs from allergic patients were in a stationary state on the first day. From second to fourth day, the cells showed rapid growth. All cells then demonstrated a lag phase and reached confluency over the following days. The patterns of proliferation were similar to those of hTMSCs derived from non-allergic patients (Fig 4A) and there were no statistically significant differences in proliferation rate after TLR priming (TLR3 and TLR4) during seven days (Fig 4B). These results showed that the patterns of proliferation were consistent regardless of allergic state or TLR priming.

### Multilineage differentiation potential of hTMSCs derived from allergic and non-allergic patients

**Fig 5:**
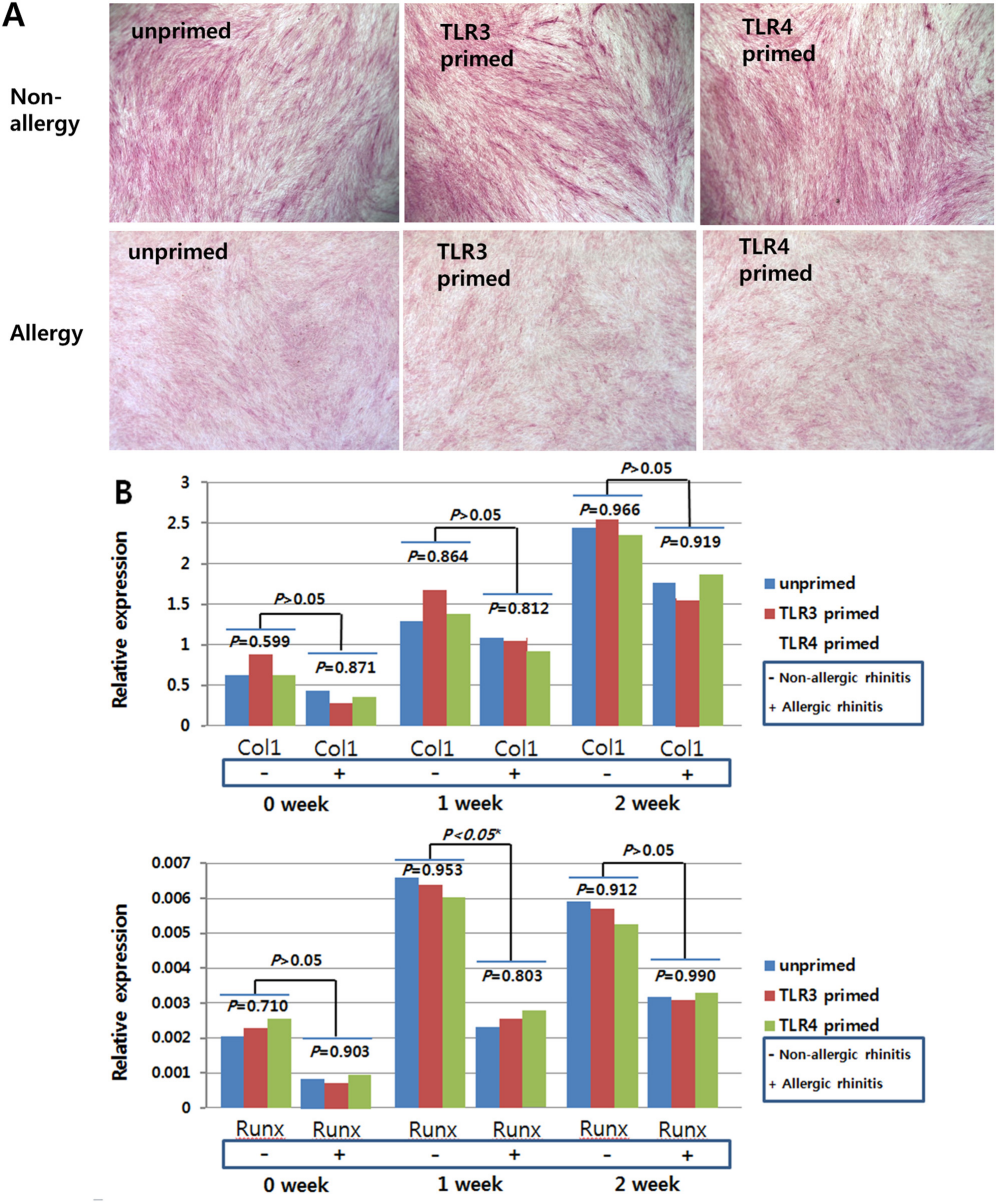
Osteogenic differentiation potential of human turbinate-derived mesenchymal stem cells (hTMSCs) from allergic and non-allergic patients. Cells were cultured in osteogenic induction medium. Alkaline phosphatase staining (× 400) on cultured hTMSCs from non-allergic patients (above) and allergic patients (below) demonstrated similar levels of staining by visual assessment. The mRNA expression of type I collagen and Runt-related transcription factor 2 (RUNX2) in hTMSCs (B) was determined by RT-PCR. hTMSCs displayed consistently increasing expression of osteogenic differentiation markers irrespective of allergic state or priming with TLR agonists.

**Fig 6:**
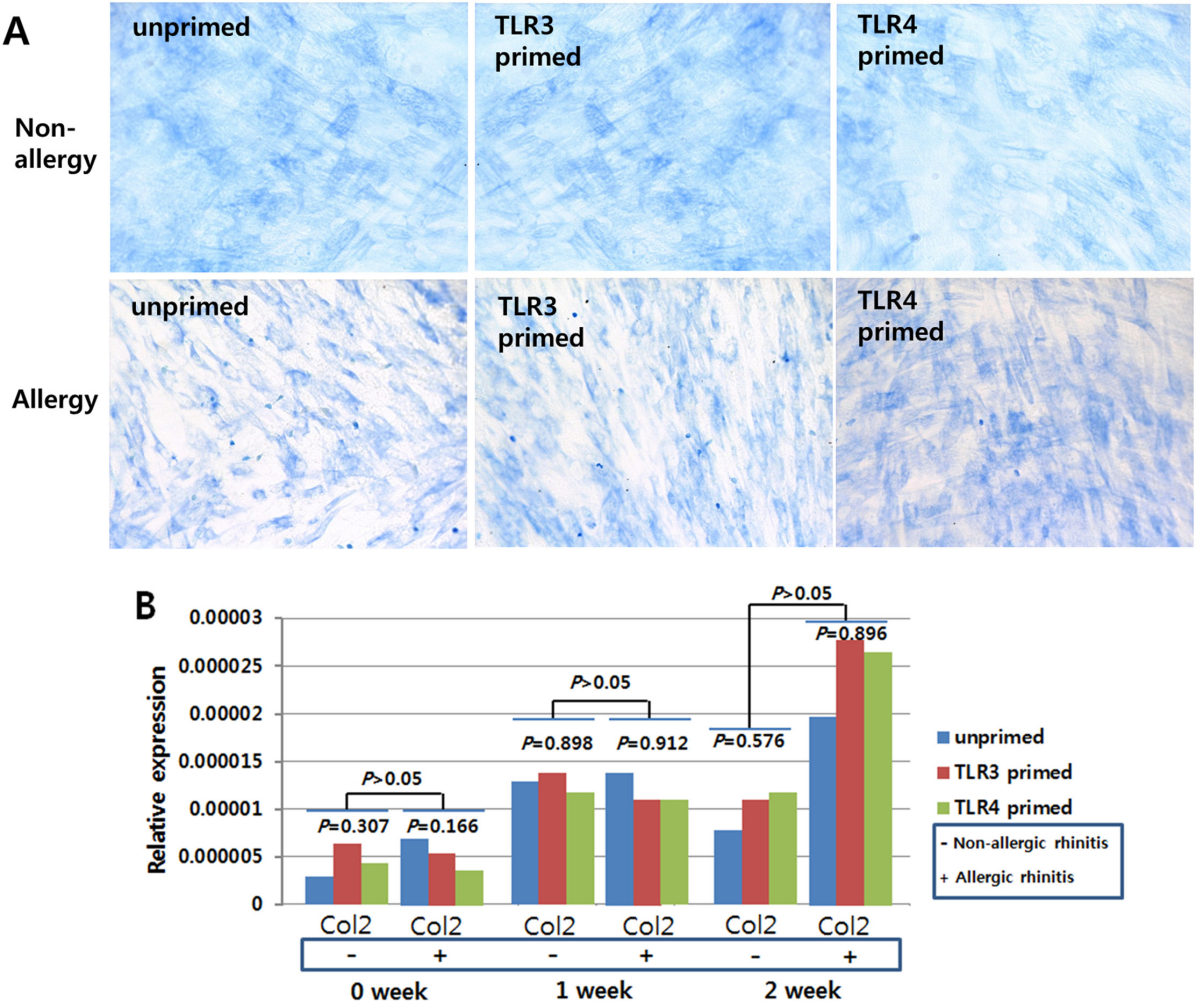
Chondrogenic differentiation potential of human turbinate-derived mesenchymal stem cells (hTMSCs) from allergic and non-allergic patients. Cells were cultured in chondrogenic induction medium. Toluidine blue staining (× 400) on cultured hTMSCs from non-allergic patients (above) and allergic patients (below) demonstrated similar amounts of staining by visual assessment. The mRNA expression of type II collagen in hTMSCs (B) was detected by RT-PCR. hTMSCs displayed consistently increasing expression of chondrogenic differentiation markers irrespective of allergic state or priming with TLR agonists.

**Fig 7:**
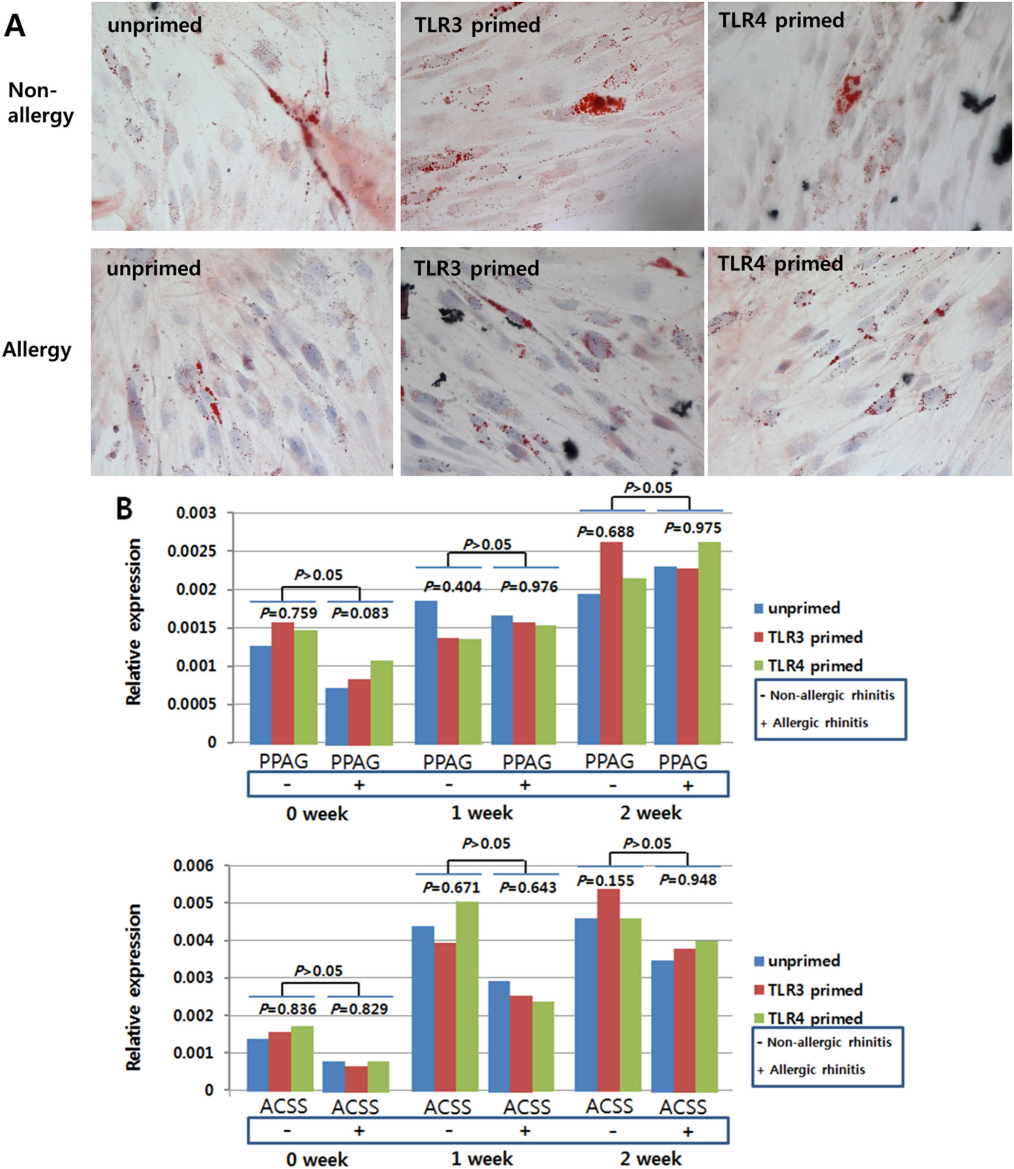
Adipogenic differentiation potential of human turbinate-derived mesenchymal stem cells (hTMSCs) from allergic and non-allergic patients. Cells were cultured in adipogenic induction medium (A). Adipogenesis was detected by the staining of intracytoplasmic lipid droplets with Oil Red O after two weeks of culture. Visual assessment demonstrated that hTMSCs from non-allergic patients (above) and allergic patients (below) displayed similar amounts of Oil Red O staining. RT-PCR analysis of peroxisome proliferator-activated receptor γ (PPARγ) and AcylCoA synthetase (ACS) mRNA expression in hTMSCs displayed consistently increasing expression of adipogenic differentiation markers irrespective of allergic state or priming with TLR agonists.

To investigate the effect of the allergic condition of donor and TLR activation on the tri-lineage (cartilage, bone, fat) differentiation capabilities of hTMSCs, we measured these differentiation capabilities according to allergic state or TLR priming. In the hTMSCs derived from allergic patients, cells in osteogenic medium displayed direct evidence of calcium mineralization via ALP staining (Fig 5A) and a consistently increasing expression pattern of mRNAs encoding type I collagen and RUNX2 by RT-PCR analysis (Fig 5B). Cells in chondrogenic medium showed a sulfated extracellular matrix by toluidine blue staining (Fig 6A) and mRNA expression encoding type II collagen was detected by RT-PCR (Fig 6B). Cells exposed to adipogenic differentiation medium showed an adipocytic phenotype, as evidenced by Oil Red O staining of multi-sized intracytoplasmic lipid droplets (Fig 7A), and consistently showed increasing expression of mRNAs encoding PPARγ and ACS (Fig 7B). The patterns of trilineage differentiation were similar to those of hTMSCs derived from non-allergic patients.

Based on these results, hTMSCs showed consistent trilineage differentiation potentials regardless of allergic state or TLR priming, although the expression of RUNX2 at seven days in unprimed and TLR3-primed states was significantly lower in allergic patients than in non-allergic patients.

## Discussion

The inferior turbinate is an elongated and paired structure inside the nose, covered by mucous membrane [13], which increase the surface area within the nose and help to filter and humidify inspired air [13]. In addition, the anterior part of the inferior turbinate also contributes to the formation of the nasal valve, which is located at the minimum cross-sectional area of the nasal passage [14]. Inflammation of the mucous membrane (allergic or non-allergic rhinitis) can cause inferior turbinates to swell (turbinate hypertrophy). The mucosal hypertrophy of the inferior turbinate significantly increases nasal airway resistance, contributing greatly to symptoms of nasal airway obstruction [15].

Partial turbinectomy, in which the anterior part of enlarged inferior turbinate is removed, is effective procedures for relieving obstruction of the nasal valve [16]. Previously, we demonstrated the inferior turbinate tissues removed during turbinate surgery could be used as a source of MSCs. Additional studies has been performed to evaluate the quality of hTMSCs and their suitability for clinical applications. Previous studies have shown that the yield of hTMSCs is almost 30 times higher than that of AD and BM-MSCs, and their proliferation rate is almost five times higher than that of BM-MSCs. The cells in 1, 4, 7, 12, 16 and 20 passages were examined for the possible effect of a mid- to long term cell culture in morphological and immunophenotypical characterization of hTMSCs. Unlike other human MSCs, there are similar morphology and comparable pattern of the expression of specific surface proteins among all six different passage groups [6]. Additionally, it has been shown that the donor age do not influence the properties of hTMSCs [7]. These data support the development of hTMSCs as an effective tool for tissue regeneration, with a further advantage being the availability of large quantities of tissue that would otherwise be discarded.

AR is characterized by dysregulation of the immune response to allergens and airway inflammation [2]. In a previous histological study, AR demonstrated a selective increase in CD4+ T cells, CD3+ T cells, B cells, and γδ T cells in the nasal mucosa and increased levels of eosinophils and mast cells within the submucosa, independent of levels in peripheral blood, compared with non-allergic rhinitis [17, 18]. These findings show that allergy is a highly complex process that affects both immune cells and the surrounding tissue, leading to a general alteration of local tissue function. This suggests that the functional properties of hTMSCs could be affected by the allergic state of the donor. However, no studies have currently revealed the characteristics of human MSCs derived from the other sources as well as turbinate associated with allergic state. Considering that the prevalence of AR reported in epidemiologic studies of various countries ranges from 10% to 25% of the general population, with increasing prevalence over the last decade [1], it is critical to understand the effects of allergy on hTMSCs if they are to be successfully used as therapeutics in regenerative medicine.

In this study, we compared the characteristics of hTMSCs from five allergic patients with those from five non-allergic patients. We evaluated levels of MSC surface markers and TLRs by FACS, and assayed the proliferation, trilineage differentiation, and immunomodulatory function (cytokine secretion) of the hTMSCs. By FACS analysis, hTMSCs from allergic and non-allergic patients demonstrated a phenotype characteristic of MSCs, and there was no significant difference between the groups. MSC surface protein markers are widely used to isolate, enrich, and characterize MSCs *in vivo* and *in vitro*. Therefore, this information is important for the analysis of MSCs numbers and characteristics regardless of donor allergy, if MSCs are isolated using one or more MSC markers [19]. Our findings demonstrate that these markers are dependable for characterizing hTMSCs in studies regarding the influence of allergy. Additionally, the expression levels and response of TLRs, specifically TLR2, TLR3, TLR4, and TLR5, were similar in the allergic and non-allergic groups. TLRs are key molecules bridging both innate and adaptive immune responses to a broad variety of antigens, and the association between TLRs and allergic diseases has been focused in many animal and human studies [20]. Fransson et al. showed an increase in protein expression for TLR2, TLR3, and TLR4 in the nasal mucosa of allergic patients and suggested a possible involvement of these TLRs in allergic airway inflammation [21]. They commented that the increase in TLR mRNA expression mainly occurred in the epithelial cells rather than the submucosal cells. By contrast, Renkonen et al. reported that baseline epithelial expression of TLR proteins was identical in healthy controls and allergic group and TLR mRNA expression was not affected by allergen challenge. Regarding these results, they explained that these differences could in part be caused by the fact that whole biopsies including the epithelium and the submucosa were used instead of epithelial cells [22]. Considering that hTMSCs are located in the submucosa but not in the epithelium, previous studies could explain our results that the expression levels and response of TLRs of hTMSCs was not affected by allergic state of donor.

In a previous study [12], although TLR4-primed hTMSCs proliferated more rapidly during the fast growth period than unprimed or TLR3-primed hTMSCs in non-allergic patients, the *p*-value for the difference in proliferation rate in the previous study was marginal (0.04) and the growth curve showed the similar proliferation patterns regardless of TLR priming. Additionally, the current result showed that the exposure to TLR agonists had no significant effect on the proliferation of the allergic and non-allergic groups over a period of seven days. These results could mean that TLR activation may not significantly affect the proliferating ability of hTMSCs and the differences could be explained by variability between individual donors. Although MSCs have been reported to ameliorate allergic airway inflammation by suppressing the activation or proliferation of T lymphocyte or inducing regulatory T Cells in allergic models, there has been no report regarding the effect of local allergic state on the proliferation of MSCs [23, 24]. The proliferation patterns of hTMSCs from allergic patients were similar to those of MSCs derived from non-allergic patients. These findings support the hypothesis that allergic state of donor may not affect the proliferating ability of existing or transplanted hTMSCs *in vivo*.

Increasing evidence demonstrates that MSCs have potent immunomodulatory properties as well as reparative functions in the inflammatory niche [25]. In this study, hTMSCs secreted high amounts of IL-6 and IL-8, but not IL-10, TNF-α, or IFN-γ, irrespective of allergy state. The TLR4 priming significantly increase expression of IL-6, IL-8, GM-CSF, IP-10, and RANTES in hTMSCs irrespective of allergy state, while the TLR3 priming did not significantly affect the secretion of these cytokines. These results were similar to the results of a previous study [12]. Although upregulation of the cytokines IL-12 and TNF-α following TLR4 agonist exposure was only seen in hTMSCs from the non-allergic group, this difference was not definitive because the levels of these cytokines were very low (almost 1 pg/ml) [12]. Based on our results, we suggested that allergic state of donor would not affect the responsiveness of hTMSCs to TLR agonists. However, the baseline level of cytokines, except RANTES, was higher in the allergic group than in the non-allergic group, although this difference was not statistically significant. Previously, we suggested that hTMSCs play a pivotal role in both initiating the clearance of pathogens and promoting the repair of injured tissue [12, 26, 27]. Although we could not verify this, these differences are probably responsible for the fact that turbinate tissue possesses different histology and immune status in the allergic state, and persistently contact large amounts of allergen, which makes it necessary for the hTMSCs to secrete the relatively different baseline amounts of cytokines.

We measured the effect of allergy on the trilineage (bone, cartilage, and adipose tissue) differentiation of hTMSCs via histological staining and gene expression levels. During osteogenic, chondrogenic, and adipogenic differentiation of hTMSCs, the intensity of histological staining increased in both groups. No significant differences in staining were observed by visual assessment of the two groups. The expression levels of the trilineage-specific genes (type II collagen, RUNX2, type I collagen, PPARγ, and ACS) showed a consistently increasing pattern regardless of the allergic state, although type II collagen and RUNX2 expression in the non-allergic group plateaued or decreased during the second week. The expression levels of the trilineage-specific genes were not significantly different between the two groups. These results could indicate that allergy does not significantly affect the osteogenic, chondrogenic, or adipogenic capacity of hTMSCs. However, the expression levels of the osteocyte and adipocyte-specific genes tended to be higher in the non-allergic group than in the allergic group. RANTES is an adipokine that is upregulated in adipose tissue by obesity in both mice and humans [28], and is important for inducing osteogenesis of human MSCs [29]. The baseline and activated levels of RANTES were lower in the allergic group than in the non-allergic group, which could explain these tendencies, although there is still a lack of knowledge of hTMSC mechanisms in the allergic state.

This study is the first to demonstrate that hTMSCs express MSC-specific surface proteins, are highly proliferative, and differentiate into cells with the trilineage phenotype irrespective of the allergic state of the donor. Considering the high and increasing incidence of AR, these results suggest that the allergic state of donor would not affect the clinical use of autologous or allogenic hTMSCs. However, although the harvest of MSCs after allergen challenge in the allergic patient and comparison with these MSCs may be ideal for the evaluation of the characteristics of hTMSCs regarding allergic response, this method could not be performed in consideration with medical ethics. In addition, this was not an experimental study which can gather subjects for the purpose of study and control all cofounders because all subjects enrolled in this study came or were transfered to our hospital for the diagnosis and treatment of suspicious allergic rhinitis. Nevertheless, because the harvest of hTMSCs after allergen provocation would not be very frequent in practice, the results described here provide important information regarding a novel source of multipotent MSCs.

## Conclusion

These data suggest that the allergic state of donor does not influence the characteristics, proliferation, or differentiation potential of hTMSCs, allowing the use of a wider range of multipotent MSC donors. This will help development of an efficient way for tissue regeneration due to the availability of large quantities of tissue that were previously removed. Moreover, these results are informative for the establishment of custom tissue and cell banks for patients with various diseases.

## Supporting Information

S1 Table The values of toll-like receptor (TLR) expression and response to TLR agonists from allergic and non-allergic patients. (DOCX)

S2 Table The values of cytokine secretion by human turbinate-derived mesenchymal stem cells (hTMSCs) from allergic and non-allergic patients according to the treatments of toll-like receptor (TLR) agonists. (DOCX)

S3 Table The values of cell proliferation according to the treatments of toll-like receptor (TLR) agonist in human turbinate-derived mesenchymal stem cells (hTMSCs) from allergic and non-allergic patients. (DOCX)

S4 Table The values of mRNA expression of type I collagen and Runt-related transcription factor 2 (RUNX2) of human turbinate-derived mesenchymal stem cells (hTMSCs) from allergic and non-allergic patients. (DOCX)

S5 Table The values of mRNA expression of type II collagen of human turbinate-derived mesenchymal stem cells (hTMSCs) from allergic and non-allergic patients. (DOCX)

S6 Table The values of mRNA expression of peroxisome proliferator-activated receptor γ (PPARγ) and AcylCoA synthetase (ACS) of human turbinate-derived mesenchymal stem cells (hTMSCs) from allergic and non-allergic patients. (DOCX)
