# Supplementary material for: Characteristics of Human Turbinate-Derived Mesenchymal Stem Cells Are Not Affected by Allergic Condition of Donor
Source: PLoS One. 2015 Sep 16;10(9):e0138041. doi: 10.1371/journal.pone.0138041 (PMC4574043; doi:10.1371/journal.pone.0138041)
Supplement: S1 Table — (DOCX) [file pone.0138041.s001.docx]

**S1 table. The values of toll-like receptor (TLR) expression and response to TLR agonists from allergic and non-allergic patients**

|  | TLR2 | | TLR3 | | TLR4 | | TLR5 | |
| --- | --- | --- | --- | --- | --- | --- | --- | --- |
| MAST | Negative | Positive | Negative | Positive | Negative | Positive | Negative | Positive |
|  | M (SD) | M (SD) | M (SD) | M (SD) | M (SD) | M (SD) | M (SD) | M (SD) |
| Unprimed | 3.996 (0.83368) | 3.496 (1.14849) | 14.792 (0.56949) | 15.048 (0.79748) | 14.312 (1.44391) | 14.586 (1.04433) | 5.788 (1.39067) | 5.056 (1.57001) |
| TLR3 primed | 4.49 (1.09462) | 4.764 (0.60302) | 14.644 (0.51340) | 14.64 (0.83774) | 14.55 (1.61693) | 14.866 (1.67967) | 5.26 (1.09515) | 4.368 (0.84165) |
| TLR4 primed | 6.502 (1.92606) | 6.326 (1.07398) | 15.454 (0.57440) | 15.406 (0.58385) | 17.59 (0.93226) | 17.664 (1.13434) | 7.352 (1.93802) | 5.606 (1.62803) |

Abbreviation: M, mean; SD, standard deviation
