# Supplementary material for: Characteristics of Human Turbinate-Derived Mesenchymal Stem Cells Are Not Affected by Allergic Condition of Donor
Source: PLoS One. 2015 Sep 16;10(9):e0138041. doi: 10.1371/journal.pone.0138041 (PMC4574043; doi:10.1371/journal.pone.0138041)
Supplement: S2 Table — (DOCX) [file pone.0138041.s002.docx]

**S2 table. The values of cytokine secretion by human turbinate-derived mesenchymal stem cells (hTMSCs) from allergic and non-allergic patients according to the treatments of toll-like receptor (TLR) agonists**

|  | IL4 | | GMCSF | | IFNr | | IL10 | |
| --- | --- | --- | --- | --- | --- | --- | --- | --- |
| MAST | Negative | Positive | Negative | Positive | Negative | Positive | Negative | Positive |
|  | M (SD) | M (SD) | M (SD) | M (SD) | M (SD) | M (SD) | M (SD) | M (SD) |
| Unprimed | 1.35725 (0.770686) | 1.722259 (0.575642) | 0.6852 (0.088296) | 1.6648 (0.519121) | 0.63125 (0.398433) | 1.31314 (1.794059) | 3.784 (3.640540) | 7.44486 (3.465675) |
| TLR3 primed | 1.14475 (0.401277) | 1.3425 (0.650459) | 0.9008 (0.474385) | 1.0655 (0.612521 | 0.63267 (0.166854) | 0.50117 (0.202832) | 3.1675 (2.514059) | 1.82371 (2.032452) |
| TLR4 primed | 1.79975 (1.202730) | 1.44557 (0.676011) | 8.162 (7.400218) | 7.26143 (7.762483) | 0.8455 (0.483990) | 1.84886 (2.416493) | 3.2406 (2.892175) | 4.28286 (4.173311) |
|  | IL12p70 | | IL1a | | IL1b | | TNFa | |
| MAST | Negative | Positive | Negative | Positive | Negative | Positive | Negative | Positive |
|  | M (SD) | M (SD) | M (SD) | M (SD) | M (SD) | M (SD) | M (SD) | M (SD) |
| Unprimed | 0.6834 (0.11314504) | 2.199333 (3.9211780) | 0.93025 (0.532849) | 1.7116 (1.931359) | 0.536 (0.151718) | 1.07243 (1.416522) | 0.2912 (0.090850) | 0.51786 (0.485704) |
| TLR3 primed | 0.734 (0.0002131) | 0.6075 (0.1460696) | 1.042 (0.217835) | 0.71571 (0.275795) | 0.4004 (0.168743) | 0.51143 (0.170615) | 0.2954 (0.114071) | 0.29814 (0.104514) |
| TLR4 primed | 0.9676 (0.13930111) | 1.361 (0.8705159) | 0.7985 (0.175069) | 1.158 (0.473117) | 0.5228 (0.241584) | 0.65586 (0.272784) | 1.4084 (0.518167) | 0.65186 (0.577893) |
|  | IL6 | | IL8 | | IP10 | | RANTES | |
| MAST | Negative | Positive | Negative | Positive | Negative | Positive | Negative | Positive |
|  | M (SD) | M (SD) | M (SD) | M (SD) | M (SD) | M (SD) | M (SD) | M (SD) |
| Unprimed | 412.2 (298.328175) | 867.28571 (636.317194) | 230.8 (69.124525) | 350.71429 (234.662676) | 6.082 (2.092276) | 9.63 (8.091690) | 27.52 (15.648386) | 14.87717 (12.047331) |
| TLR3 primed | 493.4 (348.843518) | 1062.71429 (560.850757) | 254.6 (120.427572) | 404.14286 (219.761559) | 6.212 (1.646578) | 5.56 (2.208013) | 30.138 (19.550939) | 11.05429 (7.698567) |
| TLR4 primed | 1399.4 (621.477111) | 2787.14286 (368.045028) | 3244 (1677.536885) | 3458.71429 (2152.465618) | 76.88 (76.805807) | 25.06833 (18.079392) | 258.43 (166.631328) | 100.54143 (89.214422) |

Abbreviation: M, mean; SD, standard deviation
