# Supplementary material for: Characteristics of Human Turbinate-Derived Mesenchymal Stem Cells Are Not Affected by Allergic Condition of Donor
Source: PLoS One. 2015 Sep 16;10(9):e0138041. doi: 10.1371/journal.pone.0138041 (PMC4574043; doi:10.1371/journal.pone.0138041)
Supplement: S3 Table — (DOCX) [file pone.0138041.s003.docx]

**S3 table. The values of cell proliferation according to the treatments of toll-like receptor (TLR) agonist in human turbinate-derived mesenchymal stem cells (hTMSCs) from allergic and non-allergic patients.**

| Day | 0 | 1 | 2 | 3 | 4 | 5 | 6 | 7 |
| --- | --- | --- | --- | --- | --- | --- | --- | --- |
| MAST | Negative | | | | | | | |
|  | M (SD) | M (SD) | M (SD) | M (SD) | M (SD) | M (SD) | M (SD) | M (SD) |
| Unprimed | 0.5366 (0.099334) | 0.6769 (0.288571) | 1.0553 (0.358524) | 1.6634 (0.656059) | 2.4422 (0.741141) | 2.9951 (0.529471) | 3.3071 (0.270607) | 3.4376 (0.245379) |
| TLR3 primed | 0.5574 (0.046463) | 0.757 (0.289983) | 1.3925 (0.590445) | 2.0533 (1.137613) | 2.6704 (0.922875) | 3.2501 (0.554107) | 3.4119 (0.352615) | 3.6219 (0.343130) |
| TLR4 primed | 0.5658 (0.077928) | 0.8994 (0.507875) | 1.7745 (1.121612) | 2.24 (1.331169) | 2.6569 (1.004272) | 3.1876 (0.606775) | 3.4575 (0.378526) | 3.6395 (0.367175) |
| Day | 0 | 1 | 2 | 3 | 4 | 5 | 6 | 7 |
| MAST | Positive | | | | | | | |
|  | M (SD) | M (SD) | M (SD) | M (SD) | M (SD) | M (SD) | M (SD) | M (SD) |
| Unprimed | 0.37223 (0.051630) | 0.658 (0.126138) | 1.2535 (0.552526) | 1.81077 (0.707121) | 2.59861 (0.561103) | 2.75655 (0.409007) | 3.03705 (0.553785) | 3.36277 (0.279728) |
| TLR3 primed | 0.46695 (0.1800120 | 0.80368 (0.336251) | 1.14686 (0.350601) | 1.88641 (0.571159) | 2.51856 (0.680987) | 2.86045 (0.430041) | 3.08809 (0.482120) | 3.3905 (0.255377) |
| TLR4 primed | 0.39659 (0.100855) | 0.73514 (0.267789) | 1.31755 (0.767120) | 1.95618 (0.739761) | 2.71428 (0.491030) | 2.89918 (0.425061) | 3.13 (0.464482) | 3.40255 (0.295224) |

Abbreviation: M, mean; SD, standard deviation
