# Supplementary material for: Characteristics of Human Turbinate-Derived Mesenchymal Stem Cells Are Not Affected by Allergic Condition of Donor
Source: PLoS One. 2015 Sep 16;10(9):e0138041. doi: 10.1371/journal.pone.0138041 (PMC4574043; doi:10.1371/journal.pone.0138041)
Supplement: S4 Table — (DOCX) [file pone.0138041.s004.docx]

**S4 table. The values of mRNA expression of type I collagen and Runt-related transcription factor 2 (RUNX2) of human turbinate-derived mesenchymal stem cells (hTMSCs) from allergic and non-allergic patients.**

| **Type I collagen** | | | | | | |
| --- | --- | --- | --- | --- | --- | --- |
|  | 0 week | | 1 week | | 2 week | |
| MAST | Negative | Positive | Negative | Positive | Negative | Positive |
|  | M (SD) | M (SD) | M (SD) | M (SD) | M (SD) | M (SD) |
| Unprimed | 0.616577778 (0.4000187332) | 0.437122222 (0.4165550497) | 1.307722222 (0.9364168033) | 1.044677778 (0.5124581270) | 2.440133333 (1.4376085550) | 1.780344444 (1.7214734612) |
| TLR3 primed | 0.866588889 (0.8477514766) | 0.355077778 (0.2703892693) | 1.5844 (1.2456897487) | 0.993944444 (0.4311730224) | 2.560411111 (1.4401957579) | 1.526188889 (1.3773384855) |
| TLR4 primed | 0.616366667 (0.0007139903) | 0.396566667 (0.0003276771) | 1.429911111 (0.0034487669) | 0.911188889 (0.0008705253) | 2.393922222 (0.0002321599) | 1.838833333 (0.0017982160) |
| **Runt-related transcription factor 2** | | | | | | |
|  | 0 week | | 1 week | | 2 week | |
| MAST | Negative | Positive | Negative | Positive | Negative | Positive |
|  | M (SD) | M (SD) | M (SD) | M (SD) | M (SD) | M (SD) |
| Unprimed | 0.001966333 (0.0013860442) | 0.000870222 (0.0007088206) | 0.006619111 (0.0046659816) | 0.002448889 (0.0015518009) | 0.005794444 (0.00329358510 | 0.003142333 (0.0032336526) |
| TLR3 primed | 0.002343222 (0.0015547398) | 0.000836 (0.0006727553) | 0.006333889 (0.0043835465) | 0.002697778 (0.0015816509) | 0.005765556 (0.0032835618) | 0.003098667 (0.0028053068) |
| TLR4 primed | 0.002676889 (0.0023431551) | 0.000913778 (0.0007802474) | 0.005981 (0.0039941810) | 0.002947778 (0.0016408289) | 0.005251111 (0.0023498215) | 0.003295444 (0.0030379509) |

Abbreviation: M, mean; SD, standard deviation
