# Supplementary material for: Characteristics of Human Turbinate-Derived Mesenchymal Stem Cells Are Not Affected by Allergic Condition of Donor
Source: PLoS One. 2015 Sep 16;10(9):e0138041. doi: 10.1371/journal.pone.0138041 (PMC4574043; doi:10.1371/journal.pone.0138041)
Supplement: S5 Table — (DOCX) [file pone.0138041.s005.docx]

**S5 table. The values of mRNA expression of type II collagen of human turbinate-derived mesenchymal stem cells (hTMSCs) from allergic and non-allergic patients.**

| **Type II collagen** | | | | | | |
| --- | --- | --- | --- | --- | --- | --- |
|  | 0 week | | 1 week | | 2 week | |
| MAST | Negative | Positive | Negative | Positive | Negative | Positive |
|  | M (SD) | M (SD) | M (SD) | M (SD) | M (SD) | M (SD) |
| Unprimed | 0.000003085 (0.0000026467) | 0.000006497 (0.0000221315) | 0.00001266 (0.0000077106) | 0.000013064 (0.0000168167) | 0.000008197 (0.0000063781) | 0.000019904 (0.0000265832) |
| TLR3 primed | 0.000006343 (0.0000057937) | 0.000005359 (0.0000068053) | 0.000013327 (0.0000039203) | 0.000010583 (0.0000137346) | 0.000010499 (0.0000054436) | 0.000027137 (0.0000374344) |
| TLR4 primed | 0.000004536 (0.0000041816) | 0.000004241 (0.0000051693) | 0.000011969 (0.0000063328) | 0.000010165 (0.0000130004) | 0.000010808 (0.0000051893) | 0.000026223 (0.0000356471) |

Abbreviation: M, mean; SD, standard deviation
