# Supplementary material for: Characteristics of Human Turbinate-Derived Mesenchymal Stem Cells Are Not Affected by Allergic Condition of Donor
Source: PLoS One. 2015 Sep 16;10(9):e0138041. doi: 10.1371/journal.pone.0138041 (PMC4574043; doi:10.1371/journal.pone.0138041)
Supplement: S6 Table — (DOCX) [file pone.0138041.s006.docx]

**S6 table. The values of mRNA expression of peroxisome proliferator-activated receptor γ (PPARγ) and AcylCoA synthetase (ACS) of human turbinate-derived mesenchymal stem cells (hTMSCs) from allergic and non-allergic patients.**

| **Peroxisome proliferator-activated receptor r** | | | | | | |
| --- | --- | --- | --- | --- | --- | --- |
|  | 0 week | | 1 week | | 2 week | |
| MAST | Negative | Positive | Negative | Positive | Negative | Positive |
|  | M (SD) | M (SD) | M (SD) | M (SD) | M (SD) | M (SD) |
| Unprimed | 0.00133225 (0.0004983202) | 0.000728857 (0.0002558440) | 0.001819625 (0.0010787299) | 0.001686714 (0.0008435194) | 0.001965 (0.0011390221) | 0.002338286 (0.0020591799) |
| TLR3 primed | 0.00151975 (0.0007318795) | 0.000811286 (0.0002951202) | 0.00142 (0.0001155113) | 0.001600286 (0.0010316339) | 0.0025875 (0.0011980669) | 0.002327143 (0.0021063216) |
| TLR4 primed | 0.0014825 (0.0002522329) | 0.001054 (0.0002375710) | 0.0014015 (0.0004894288) | 0.001583571 (0.0009353131) | 0.002131375 (0.0019551085) | 0.002559143 (0.0022915360) |
| **AcylCoA synthetase** | | | | | | |
|  | 0 week | | 1 week | | 2 week | |
| MAST | Negative | Positive | Negative | Positive | Negative | Positive |
|  | M (SD) | M (SD) | M (SD) | M (SD) | M (SD) | M (SD) |
| Unprimed | 0.00151875 (0.0007054417) | 0.000792857 (0.0004618768) | 0.0042875 (0.0017347602) | 0.002964286 (0.0009301587) | 0.00462875 (0.0003713850) | 0.00368 (0.0015916344) |
| TLR3 primed | 0.001602625 (0.0007702391) | 0.000675714 (0.0003805705) | 0.00394625 (0.0016132924) | 0.002581429 (0.0012677990) | 0.00525875 (0.0011660977) | 0.00381 (0.0017140984) |
| TLR4 primed | 0.00173625 (0.0007139903) | 0.000782429 (0.0003276771) | 0.0050125 (0.0034487669) | 0.002458571 (0.0008705253) | 0.00463125 (0.0002321599) | 0.003978571 (0.0017982160) |

Abbreviation: M, mean; SD, standard deviation
